# Long-Term Outcomes of Percutaneous Cryoablation for Patients with Hepatocellular Carcinoma within Milan Criteria

**DOI:** 10.1371/journal.pone.0123065

**Published:** 2015-04-07

**Authors:** Guanghua Rong, Wenlin Bai, Zheng Dong, Chunping Wang, Yinying Lu, Zhen Zeng, Jianhui Qu, Min Lou, Hong Wang, Xudong Gao, Xiujuan Chang, Linjing An, Hongyan Li, Yan Chen, Ke-Qin Hu, Yongping Yang

**Affiliations:** 1 Center of Therapeutic Research for Liver Cancer, the 302 Hospital, 100 Xi Si Huan Middle Road, Beijing 100039, China; 2 Division of Gastroenterology/Hepatology, University of California, Irvine, 101 the City Dr., Building 56, Ste. 237, Orange, CA 92868, United States of America; National Yang-Ming University, TAIWAN

## Abstract

**Background:**

Accumulating evidences have suggested that percutaneous cryoablation could be a valuable alternative ablation therapy for HCC but there has been no large cohort-based analysis on its long-term outcomes.

**Methods:**

A series of 866 patients with Child-Pugh class A-B cirrhosis and HCC within Milan criteria who underwent percutaneous cryoablation was long-term followed. The safety, efficacy, 5-year survival, and prognostic factors of percutaneous cryoablation in the treatment of HCC were analyzed.

**Results:**

A total of 1197 HCC lesions were ablated with 1401 cryoablation sessions. Complete response (CR) was achieved in 1163 (97.2%) lesions and 832 (96.1%) patients with 34 (2.8%) major complications, but no treatment-related mortality. After a median of 30.9 months follow-up, 502 (60.3%) patients who achieved CR developed different types of recurrence. The cumulative local tumor recurrence rate was 24.2% at 5-years. Multiple tumor lesions, tumor size > 3 cm, and repeated ablation of same lesion were independent risk factors associated with local recurrence. The 5-year overall survival (OS) rates were 59.5%. Age < 36 years, HCC family history, baseline hepatitis B virus DNA >10^6^ copies/ml, and three HCC lesions were independently and significantly negative predictors to the post-cryoablation OS.

**Conclusions:**

Percutaneous cryoablation is an effective therapy for patients with HCC within Milan criteria, with comparable efficacy, safety and long-term survival to the reported outcomes of radiofrequency ablation.

## Introduction

Hepatocellular carcinoma (HCC) is the sixth most common cancer and the third leading cause of cancer-related mortality globally and the total HCC patients in China account for 55% of all cases worldwide[1–3]. Internationally endorsed guidelines currently recommend surgical resection (SR) as the first-line therapeutic option for patients with early-stage HCC and well-preserved liver function, and orthotropic liver transplantation (OLT) as the alternative option for those who are contraindicated for hepatic resection. However, the resectability rate of HCC has been limited to 20–30% because of various unfavorable factors, such as multifocal tumor lesions, underlying cirrhosis, and limited hepatic reserve as a result of decompensated cirrhosis. OLT is also not a practical option for many HCC patients due to a significant shortage of organ donors in many countries and regions[2,3]. Hence, percutaneous local ablative therapies (PLATs), including percutaneous ethanol injection (PEI), radiofrequency ablation (RFA), microwave ablation, laser ablation, and cryoablation (or cryotherapy), have been the alternative options for unresectable HCC in cirrhotic patients[2,4].

Despite being wildly used in various other cancers[5], the application of percutaneous cryoablation in HCC was sparsely reported. Compared to RFA, cryoablation endows several unique advantages including larger ablative zones, more clearly discernible treatment margin, less pain and stronger ectopic tumor suppression effects[6–8]. Over the past decade, substantial technical improvements have been achieved in cryoablation technology, including percutaneous approaches and new generation of Argon-helium Cryo-equipment with thinner probes[8], in favor of which clinical application of cryoablation in HCC have been increased significantly. For example, we have reported that the incidence of major complications and tumor seeding was as low as 6.3% and 0.78%, respectively, in patients with HCC who underwent percutaneous cryoablation[9,10]. Moreover, when compared to SR or RFA, cryoablation also showed equally good outcomes and even superior ability of local tumor control in the treatment of HCC < 5cm [11–14]. Thus, evidences have been accumulating in recent years suggesting that cryoablation could be a valuable additional therapeutic option for HCC. However, there has been no large cohort-based analysis on the long-term outcomes including safety, efficacy, 5-year survival, and prognostic factors of cryoablation in the treatment of HCC. To address this issue, we retrospectively analyzed a prospective series of 866 patients with HCC within Milan criteria who were consecutively referred for and treated with percutaneous cryoablation in our center.

## Materials and Methods

### Study concept

The present study met the requirements of the Declaration of Helsinki and was conducted via chart review, data collection and analysis. The study protocol was reviewed and approved by the institutional review board (IRB) of the 302 Hospital, Beijing, China, and written informed consent was obtained from each patient included in the study. During the study period, there were no evidence-based consensus or recommendations on selecting cryoablation vs. RFA in the treatment for cirrhotic patients with HCC meeting Milan Criteria, and therefore, both therapies plus SR, OLT and transcatheter arterial chemoembolization (TACE) were all available in our hospital as the therapeutic options for these patients. The treatment decision and plan have been made through a multidisciplinary evaluation. For those qualified for cryoablation, further discussion was provided to explain the treatment-related details and alternative options before the patient finally chose to receive cryoablation.

### Patients

Patients who met the following baseline inclusion criteria would be included to the current study: 1) Patients with HCC lesion(s) limited to Milan criteria (i.e., having a single nodule ≤5 cm in diameter or up to 3 nodules ≤3 cm in diameter[15]); 2) no extrahepatic HCC metastases, or invasion of the portal vein; 3) no prior HCC treatment; 4) underlying Child-Pugh class A or B cirrhosis; 5) no evidence of severe coagulopathy (i.e., prolonged prothrombin time of > 5 seconds) or severe thrombocytopenia (i.e., platelet count ≤ 40× 10^9^/L; 6) if ascites was diagnosed, it must be well controlled before enrollment; and 7) Eastern Cooperative Oncology Group Performance Status (ECOG PS) of 0 to 2.

Patients were excluded if any of the following conditions existed: 1) uncontrolled or refractory ascites, ongoing variceal bleeding, or encephalopathy; 2) Child-Pugh grade C cirrhosis; 3) HCC lesions beyond above inclusion, or located within 5 mm of the gallbladder, colon, stomach or the common bile duct.

Patients were diagnosed with HCC based on the criteria combining clinical, imaging, and/or histological findings[2]. Diagnosis of cirrhosis was based on 1) a long history of chronic liver disease e.g. hepatitis C virus (HCV) or hepatitis B virus (HBV) infection; 2) imaging features (evidence of nodular liver surface, caudate lobe hypertrophy, ‘‘coarse” liver tissue pattern and endoscopic findings (varices and/or portal hypertensive gastropathy), and 3) clinical evidence of portal hypertension, defined as the presence of esophagogastric varices and/or splenomegaly with platelet count lower than 100×10^9^/L, and/or history of ascites. HCC family history was defined as HCC occurred in a patient’s parents, siblings, and/or offspring.

**Fig 1:**
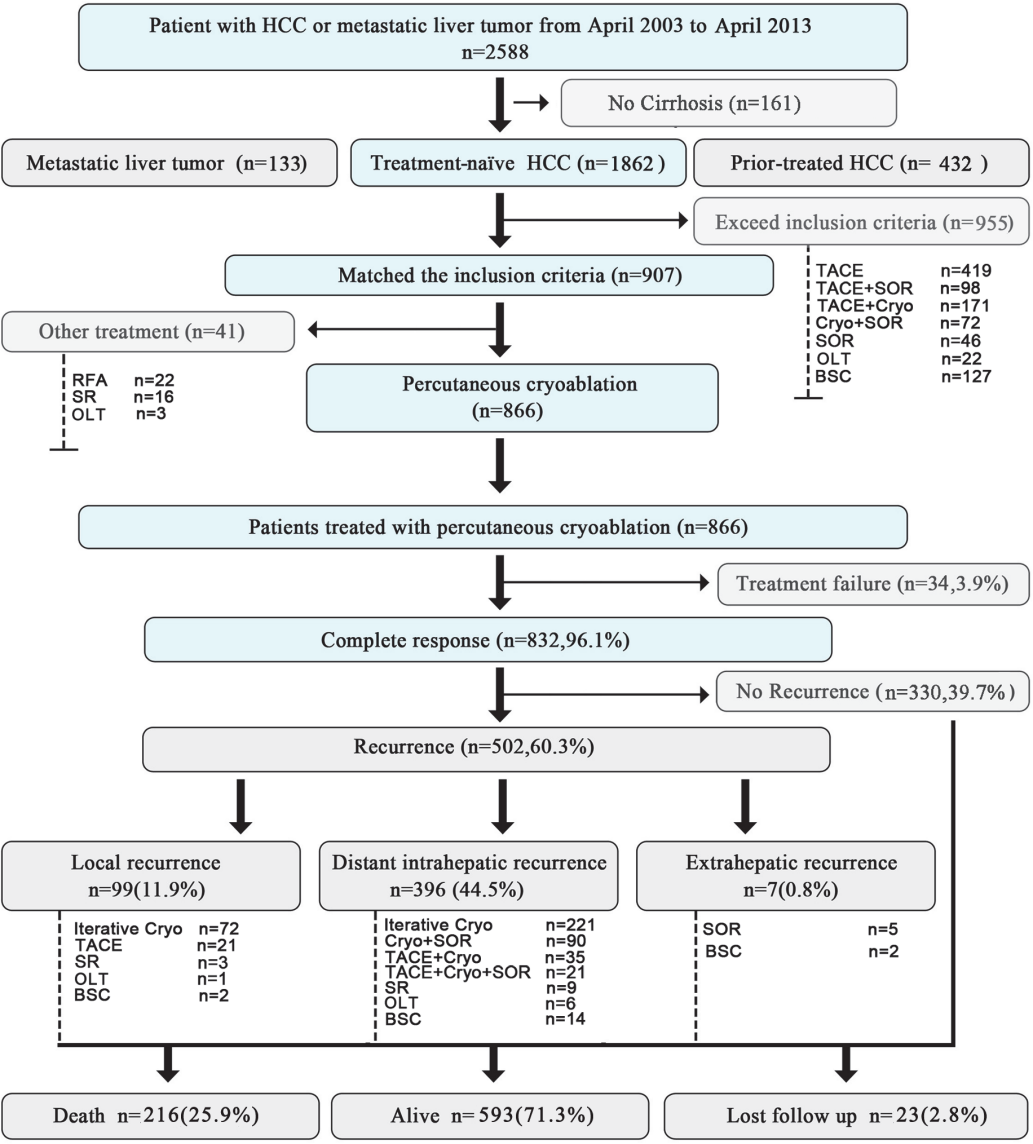
Flow chart of the present study. HCC, hepatocellular carcinoma; TACE, transarterial chemoembolization; Cryo, cryoablation; SOR, sorafenib; OLT, orthotropic liver transplantation; RFA, radiofrequency ablation; SR, surgical resection; BSC, best supportive care.

As summarized in **Fig 1**, from April 2003 to April 2013, 2,588 patients with HCC or metastatic liver tumor were consecutively referred to our Center. Among these patients, 161 (6.2%) had no cirrhosis; 432 (16.7%) had prior-treated HCC; 133 (5.1%) had metastatic liver tumors; and 1,862 (72.0%) had cirrhosis and newly diagnosed HCC. Nine hundred and fifty-five patients exceeded the inclusion criteria and received the following treatments: 419 with TACE, 98 with TACE plus sorafenib, 171 with TACE plus cryoablation, 72 with cryoablation plus sorafenib, 46 with sorafenib alone, 22 with OLT, and 127 with best supportive care (BSC). The remaining 907 patients met the inclusion criteria, of whom, 41 refused cryoablation and underwent other therapies (16 SR, 22 RFA and 3 OLT), and the remaining 866 patients received cryoablation as their initial treatment and were enrolled in the current study.

### Percutaneous cryoablation

Percutaneous cryoablation was performed as previously described[9,14]. The cryoablation equipment used was a 4 or 8-cryoprobe cryosurgery system manufactured by the Endocare Corporation (Irvine, CA, USA), with superconducting cryoprobes in 2 or 3 mm in diameter and sheath-and-guidewire technique were used. A 16-slice spiral CT was used for guidance, localization, and intraoperative real-time ultrasound was used for monitoring of the ablation procedures. After sedation and preparation, an 18-gauge×15 cm PTC needle (Hakko Co. Ltd, Osaka, Japan) was percutaneously inserted into the tumor lesion under CT guidance, followed by super-stiff guidewire and a customized coaxial catheter introducer (Cordis Corporation, Bridgewater, NJ, USA) that consists of dilation catheter and sheath, for cryoprobe insertion and instillation of hemostatic agents. An 8/11F coaxial catheter introducer was then used to introduce cryoprobe and deliver cryotherapy. The procedure might be repeated if additional treatment was required. The location, number, and size of cryoprobes were determined according to the “2 to 1” principle by Wang et al to assure cryoprobes should be placed within 1 cm from the tumor edge[16], no more than 2 cm interval between the probes. Generally, we use 1 cryoprobe in tumors ≤ 2 cm, 2 cryoprobes in tumors > 2cm but ≤ 3.5 cm and 3 cryoprobes in tumors > 3.5 cm. Once all the cryoprobes had been placed, the cryosurgery system was initiated to begin rapid freezing. The temperature of the cryoprobes was decreased to -100°C within 1 min, then further decreased and maintained between -150 ~ -160°C for 20 min. After that, the heating system was initiated to re-warm the cryoprobes and the second cycle was repeated. Once these cycles were complete, the cryoprobes were withdrawn and a hemostatic gelatin sponge was tamped into the sheath in attempt to stop bleeding and fill the sinuses, and the sheath was then removed.

### Definition of cryoablation treatment responses and complications

Contrast MRI was obtained in a week after the procedure to determine the effects of ablation, which were classified as complete response (CR) or incomplete response (IR). CR was defined as MRI detection of a non-enhanced area with necrosis/scar at the cryoablation site of the HCC lesion initially confirmed by pre-treatment baseline imaging. Patients with MRI evidence lacking CR were defined as IR and received repeat cryoablation as salvage treatment for up to three times. The above-mentioned evaluations were repeated 2 weeks after each salvage treatment. Those who failed to obtain CR after three cryoablation sessions were regarded as treatment failure (TF). In these cases, TACE and/or other treatments might be considered.

Cryoablation-related complications were assessed based on the patient’s complaints, US and laboratory findings. Major complications were defined as those that were life-threatening, resulted in substantial morbidity, or prolonged the hospital stay, and all others were considered as minor complications. Two other complications unique to cryoablation are referred to as “cryoshock” and “cryoreaction” were also intensively monitored. Cryoshock presents with multi-organ failure, severe coagulopathy, and disseminated intravascular coagulation[17]; whereas, cryoreaction, chills, fever, tachycardia, tachypnea and transient creatinine elevations. Both complications were assessed in the current study.

### Post-cryoablation follow-up and management protocol

After cryoablation, all patients were followed with serum α-fetoprotein (AFP), chest CT scans, and abdominal MRI scans every 3 months for the first year, and every 6 months thereafter to detect tumor recurrence. Local recurrence (LR) was defined as tumor recurrence within or at the periphery of the ablated lesion. Distant intrahepatic recurrence (DIR) was defined as new intrahepatic lesions that appeared in regions at least 1 cm away from the original ablated area. Extrahepatic recurrence (ER) was referred to any tumor recurrence outside the liver. Patients who developed LR with or without DIR or ER were considered local tumor progression, and patients who developed DIR and/or ER were considered distant recurrence[18].

Repetitive cryoablation was preferably considered for patients with LR or DIR. For patients with ER, molecular targeted therapies or other supportive therapies would be offered based on multidisciplinary recommendation.

### Statistical analysis

Quantitative variables were expressed as medians or means and standard deviations and qualitative variables as absolute frequencies or percentages. *P* < 0.05 was considered to be statistically significant. Long-term outcome was assessed in terms of OS. OS was defined as the interval between first cryoablation and death or the date of last follow-up. For patients who received OLT, the date of transplantation was considered to be the end of follow-up. Patients who were lost to follow-up were censored at the date of their last visit. Data analyses were performed using IBM-SPSS version 20.0 (SPSS, Chicago, IL, USA). Risk factors associating with OS or recurrence were assessed by Cox's proportional hazard model in all the variables listed in **Table 1**, those with a *P* value < 0.05 in univariate analysis were forwarded to multivariate analysis. The results of multivariate analyses were presented as a hazard ratio with 95% confidence interval (CI) and *P* values. Curves of the OS, local and distant recurrence rate were calculated by the Kaplan-Meier method.

**Table 1:** Baseline Characteristics of 866 HCC Patients Underwent Cryoablation and Were Included in the Present Study.

**Characteristics**	**Value**
**Age (Years)**	
Mean±SD (range)	53±10.34(18–85)
5%/25%/50%/75%/95% points (Years)	36/46/53/60/70
**Gender, no. (%)**	
Male/Female	714/152(82.4/17.6)
**Etiology of HCC, no. (%)**	
HBV	693(80.0)
HCV	125(14.4)
HBV-HCV	6(0.6)
Other	42(4.8)
**Liver Function Related Tests (Mean±SD)**	
Serum albumin (g/L)	36.4±5.44
Total bilirubin (μmol /L)	30.2±8.92
Cholinesterase (U/L)	4527±2010
Prothrombin activity (%)	65.1±15.3
Platelet Counts(×10^9^/L)	99.9±58.8
**AFP, no. (%)**	
< 20 ng/ml	306 (35.4)
20–400 ng/ml	287 (33.1)
> 400 ng/ml	273 (31.5)
**Child-Pugh class, no. (%)**	
A5	256 (29.6)
A6	283(32.5)
B7	231(26.8)
B8	96 (11.1)
**Lesion Number, no. (%)**	1197(100)
Patients with 1 lesion	636 (73.4)
Patients with 2 lesions	129 (14.8)
Patients with 3 lesions	101 (11.8)
**Tumor Size (cm)**	
Mean±SD (range)	2.87.±0.91 (1.2–5 cm)
0–3 cm, no. (%)	801 (66.9)
3.1–5 cm, no. (%)	396 (33.1)
**Diagnosis of HCC, no.(%)**	
Histopathology	276 (31.9)
Clinical criteria	590 (69.1)

## Results

### Patient characteristics, the post-cryoablation complete response and overall survival

The patients’ baseline characteristics were summarized in **Table 1.** All the 866 patients had cirrhosis and were treatment-naïve. The median follow-up was 30.9 months (mean, 22.6±35; range, 3.2–120 months). In 1197 HCC lesions were treated, the mean size was 2.87±0.91 cm (range, 1.2–5 cm) and HCC lesion > 3 cm was seen in 396 (33.1%) patients. Overall, 636 patients had one lesion; 129, two; and 101, three lesions. The mean number of cryoprobes used in tumors > 3 cm was 2.61 cryoprobes pre-tumor.

**Fig 2:**
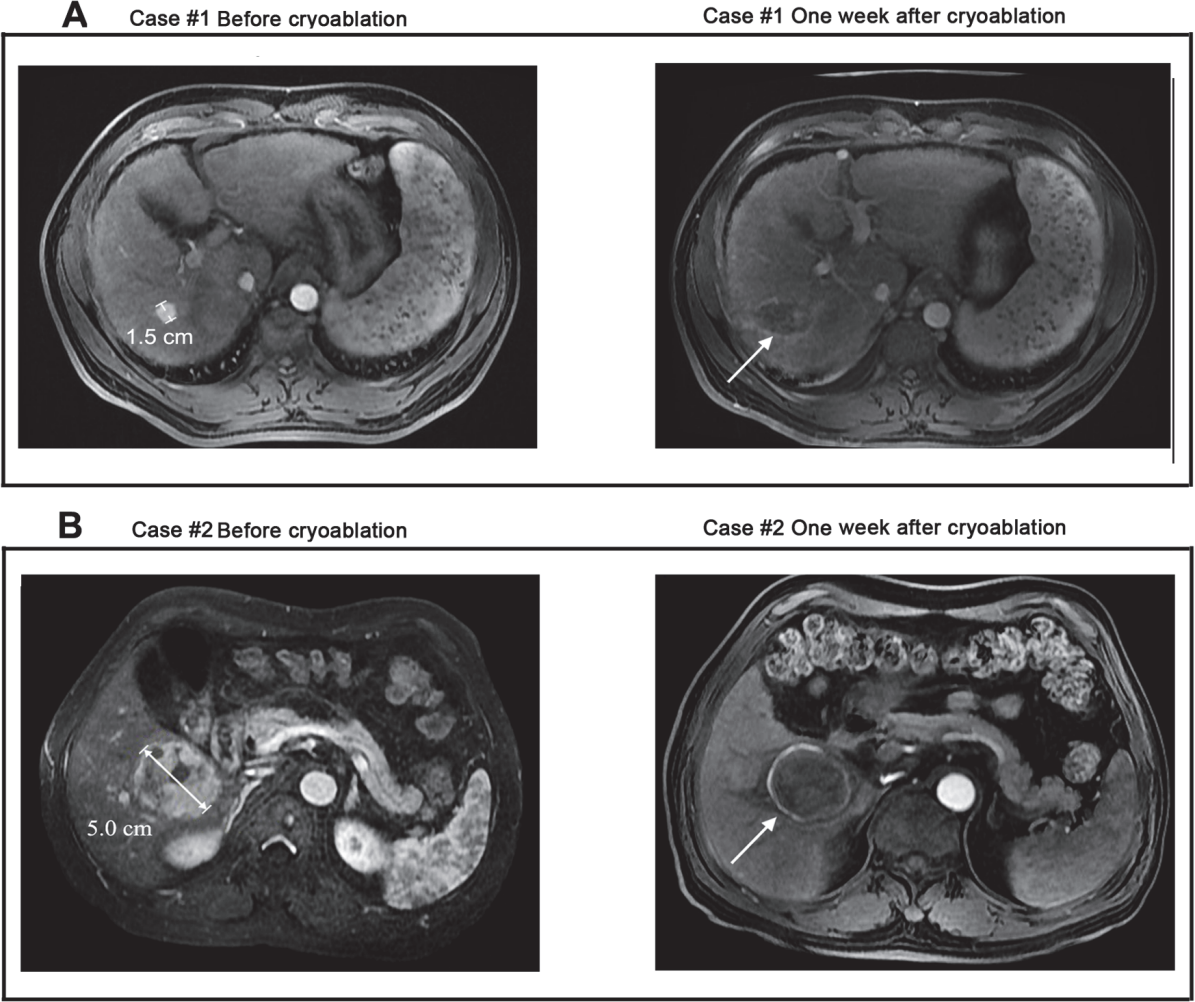
MRI images of 2 representative cases achieved CR from cryoablation. A. Case #1, a 50-year-old male with a single HCC nodule of 1.5 cm that was completely cryoablated. At the end of follow-up, he was still alive with tumor free for 120 months after treatment. **B**. Case #2, a 48-year-old female with a single HCC nodule of 5.0 cm that was completely cryoablated. At the end of follow-up, she was still alive with tumor free for >108 months after treatment.

A total of 1401 cryoablation treatments were performed for those 1197 tumor lesions: 993 (83.0%) received one; 145 (12.1%), two; and 59 (4.9%), three treatment sessions. The primary CR was achieved in 832/866 (96.1%) patients and 1163/1197 (97.2%) tumor lesions, the remaining 34/866 (3.9%) patients and 34/1197 (2.8%) residual tumor lesions were identified as TF and were excluded form further analysis. These patients were then treated with TACE (*n* = 23) or SR (*n* = 11). Images of two representative cases were shown in **Fig 2.**

At the end of follow-up, 593/832 (71.3%) cases remained survival, 216/832 (25.9%) patients died, and 23/832 (2.8%) lost follow-up. As for mortality causes, 138/216 (63.9%) were due to HCC progression; 78/216 (36.1%) were underlying cirrhosis related, including esophagogastric variceal bleeding (n = 29, 13.4%), refractory ascites-related renal failure (n = 21, 9.7%), liver failure (n = 17, 7.9%), and liver-unrelated conditions (n = 11, 5.1%). The median time from first cryoablation to death was 34.1 (mean, 35.2 ±17.5) months.

**Fig 3:**
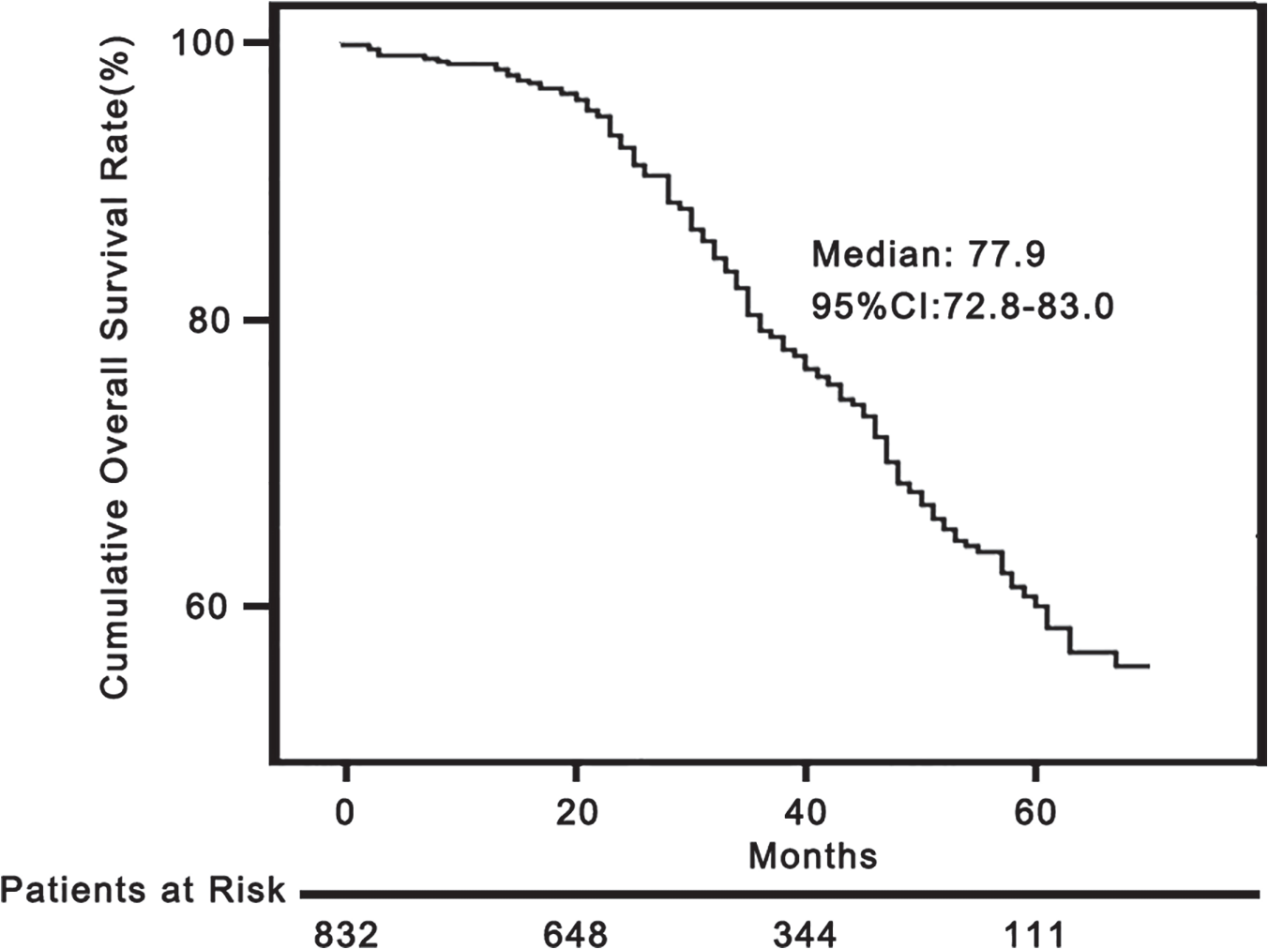
Overall survival curves of patients treated by percutaneous cryoablation. The OS curve of 832 patients who received percutaneous cryoablation as the first-line treatment.

The median post-cryoablation OS was 77.9 months (95% CI, 72.8–83.0%). The 1-, 3-, and 5-year cumulative OS rate was 98.6% (95% CI, 97.8–99.4%), 80.6% (95% CI, 77.9–83.3%), and 60.3% (95% CI, 57.2–63.4%), respectively (**Fig 3**).

A Cox’s proportional hazard model was applied to determine the risk factors associated with OS. As summarized in **Table 2**, univariate analysis indicated that 7 variables were associated with OS, while multivariate analysis confirmed that age < 36 years (*P* = 0.01), HCC family history (*P* = 0.02), baseline HBV DNA load >10^6^ copies/ml (*P* = 0.01) and three HCC lesions (*P* = 0.04) were independently and significantly negative predictors to post-cryoablation OS.

**Table 2:** Univariate and Multivariate Analyses of the Parameters that May Predict to Post-Cryoablation Overall Survival.

**Variables**	**Univariate Analysis**			**Multivariate Analysis**		
	**HR**	**95% CI**	**P**	**HR**	**95% CI**	**P**
**Age**						
> 36^‡^ yr *	1					
< 36 yr	2.21	1.28–3.80	0.01	3.02	1.57–5.82	0.01
**HCC family history**						
No *	1					
Yes	1.58	1.06–2.32	0.02	1.86	1.09–3.16	0.02
**HBV family history**						
No*	1					
Yes	1.42	1.07–1.89	0.02			
**HBV DNA copy no.**						
Non-HBV*	1					
<10^3^/ml	1.09	0.91–1.27	0.64			
10^4^-10^5^/ml	1.26	0.58–3.39	0.57			
>10^6^/ml	2.79	1.73–7.94	0.04	7.99	2.65–24.1	0.01
**Child-Pugh class**						
A *	1					
B	1.49	1.05–2.12	0.03			
**HCC Lesion** #						
1 *	1					
2	1.62	1.12–2.32	0.01			
3	2.29	1.61–3.26	0.01	1.86	1.04–2.90	0.04
**Maxim Tumor Size** ^**#**^						
≤2 cm *	1					
2.1–3.0 cm	2.43	1.02–6.01	0.06			
3.1–5.0 cm	2.99	1.22–7.35	0.02			

‡ 36 years was the 5% point of age distribution of the whole cohort.

* Reference values

# Diameter of the largest tumor as indicated by CT or MRI.

† The interval (months) form first cryoablation to first recurrence of either local or distant. Early recurrence, recurrences occurred within 24 months. Late recurrences, recurrences occurred after 24 months

Variables were analyzed by univariate model of Cox Proportional Hazard Test; those with a P-value < 0.05 were showed here and were forwarded to the multivariate analysis.

### Post-cryoablation HCC recurrence

At the end of follow-up, 502/832 (60.3%) patients who achieved CR from the initial cryoablation developed different types of HCC recurrence, including 82/502 (16.3%) with LR alone; 14/502 (2.7%), LR and DIR; 3/502 (0.6%), LR and ER; 396/502 (78.9%), DIR alone; 5/502 (1%), ER alone; and 2/502 (0.5%), DIR with ER. Overall, local HCC progression (LR or LR+DIR/ER) and distant recurrence (DIR or ER or DIR+ER) were identified in 99/832 (11.9%) and 403/832 (48.4%) patients, respectively.

**Fig 4:**
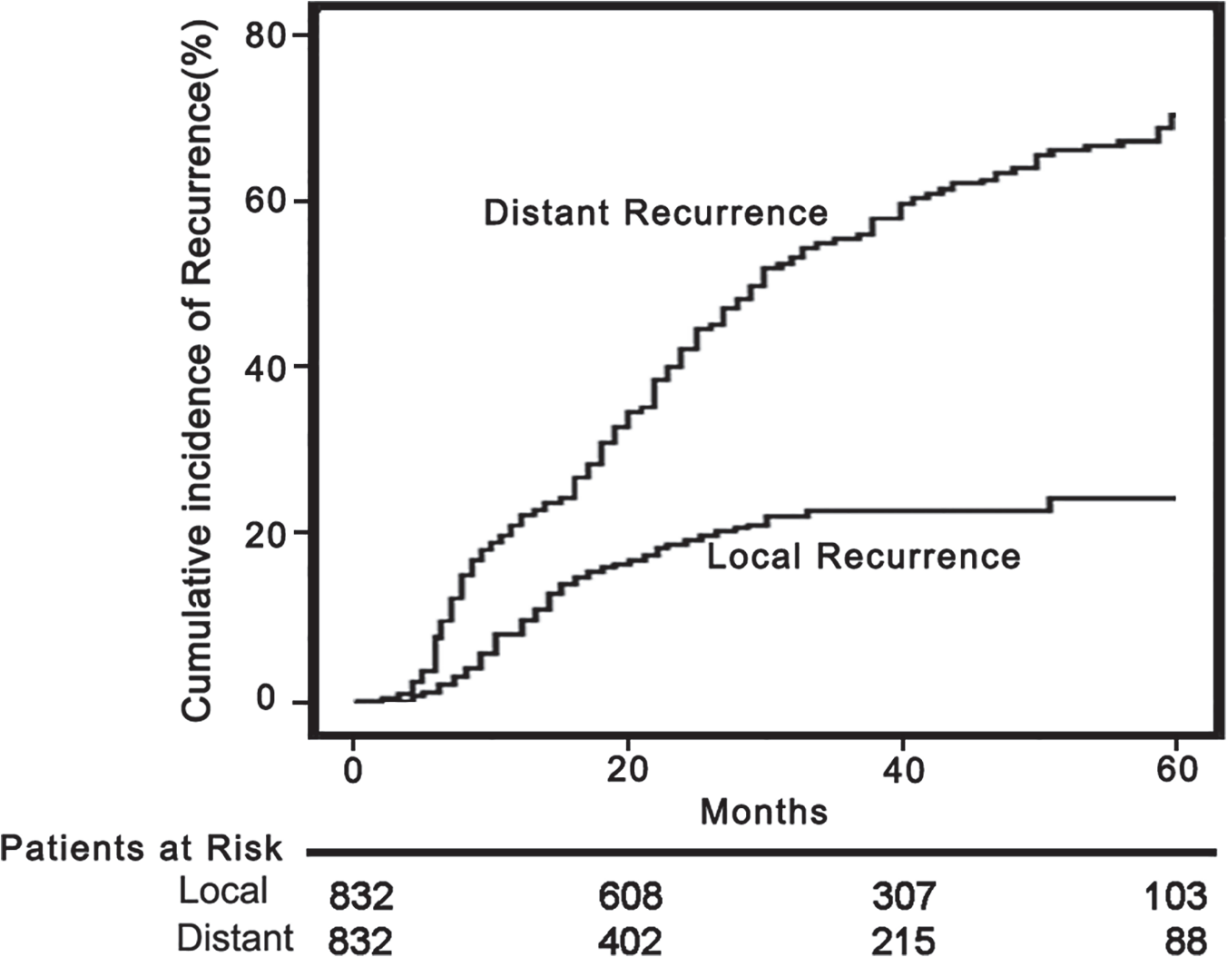
Recurrence curves of patients treated by percutaneous cryoablation. Curves of local tumor recurrence and distant tumor recurrence in 832 patients who achieved CR.

As shown in **Fig 1**, in the 502 patients with HCC recurrence, the first recurrence was treated by iterative cryoablation in 293 (58.4%), TACE in 21 (4.2%), TACE plus cryoablation in 35 (7.0%), cryoablation plus sorafenib in 90 (17.9%), TACE plus cryoablation and sorafenib in 21 (4.2%), sorafenib alone in 5 (1%), SR in 12 (2.4%), OLT in 7 (1.4%), and BSC in 18 (3.5%). The median interval from treatment to first local HCC recurrence was 21.4 months (mean, 27.1±20.4). The 1-, 3-, and 5-year rates of local HCC recurrence were 10.7% (95% CI, 9.4–11.9%), 22.1% (95% CI, 19.5–24.6%), and 24.2% (95% CI, 22.9–26.3%), respectively (**Fig 4**). The median interval from cryoablation to first distant recurrence was 24.2 months (mean, 28.1±22.3 months). The 1-, 3-, and 5-year rates of distant HCC recurrence without local tumor progression were 20.7% (95% CI, 18.9–22.5%), 48.6% (95% CI, 46.3–50.9%), and 64.9% (95% CI, 62.4–67.4%), respectively (**Fig 4**).

As shown in **Table 3**, independent risk factors associated with LR were identified as multiple tumor numbers (*P* = 0.01), tumor size >3 cm (*P* = 0.02), and repeated cryoablation to the same lesion (*P* = 0.01,). Independent risk factors associated with distant recurrence were multiple tumor numbers (*P* = 0.01), tumor size >3 cm (*P* = 0.01) and PLT count lower than 100×10^9^/L (*P* = 0.04).

**Table 3:** Analyses of the Parameters that May Predict to Post-Cryoablation HCC Recurrence.

**Variables**	**Univariate**					**Multivariate**		
	**HR**	**95% CI**	**P**			**HR**	**95% CI**	**P**
**Post-Cryoablation Local Recurrence**								
**HCV Infection**								
No *	1							
Yes	2.11	1.11–4.02	0.02					
**Tumor no.**								
1 *	1							
2	1.51	0.98–2.33	0.04			3.02	1.80–5.06	0.01
3	1.83	1.17–2.86	0.01			3.51	2.05–6.02	0.01
**Maxim Tumor Size** ^**#**^								
≤2 cm *	1							
2.1–3.0 cm	1.25	0.92–3.02	0.07					
3.1–5.0 cm	2.75	1.96–4.34	0.03			2.53	1.87–3.43	0.02
**Total Ablation times**								
1*	1							
2	1.97	1.33–2.93	0.01			1.66	1.09–2.53	0.02
3	6.44	4.05–10.24	0.01			4.67	2.86–7.64	0.01
**Post-Cryoablation Distant Recurrence**								
**Tumor no.**								
1 *	1							
2	1.14	0.96–1.49	0.04			1.53	1.09–2.13	0.01
3	1.31	1.07–1.77	0.03			1.76	1.26–2.50	0.01
**Maxim Tumor Size** ^**#**^								
≤2 cm *	1							
2.1–3.0 cm	1.43	0.87–2.35	0.16					
3.1–5.0 cm	1.80	1.09–2.97	0.02			1.93	1.15–3.26	0.01
**Total Ablation times**								
1*	1							
2	1.24	0.96–1.59	0.10					
3	1.65	1.03–2.63	0.04					
**PLT**								
>100×10^9^/L *	1							
≤100×10^9^/L	1.47	0.96–1.99	0.03			1.35	0.94–1.76	0.04

* Reference values.

^#^ Diameter of the largest tumor as indicated by CT or MRI.

Variables were analyzed by a univariate model of Cox Proportional Hazard Test; those with a P-value < 0.05 were showed here and were forwarded to the multivariate analysis.

### Cryoablation-related complications

A total of 1197 HCC lesions were ablated with 1401 CRA sessions. As summarized in **Table 4**, the major complications occurred in 29 (2.4%); and minor complications, in 128 (10.6%). There was no death directly related to cryoablation treatment or within 90 days (perioperative). None of the patients in our cohort manifested typical cryoshock. Nevertheless, cryoreaction were observed in 7 patients (0.6%) in cryoablation sessions. In our study, all these 7 patients were fully recovered within 72 hours with supportive medical management.

**Table 4:** Summary of the Complications from 1197 Cryoablation-treated HCC Lesions.

Major Complications	Number, (%)
Tumor seeding	8(0.67%)
Cryorecation	7(0.58%)
Hepatic Hemorrhage	4(0.32%)
Liver abscess	2(0.16%)
Pleural abscess	1(0.08%)
Hemothorax requiring drainage	1(0.08%)
Pleural effusion requiring drainage	6(0.50%)
Total	29(2.4%)
**Minor Complications**	
Postoperative pain	33(2.7%)
Postoperative fever	26(2.1%)
Self limiting pleural effusion	21(1.8%)
Self limiting Pneumothorax	5(0.4%)
Transient elevation of aminotransferase	12(1%)
Skin frostbite	9(0.8%)
Stress ulcer	5(0.4%)
Bleeding at the probe-inserting point	17(1.4%)
Total	128(10.6%)

## Discussion

The present study was a retrospective study on a series of 866 cirrhotic patients with HCC meeting Milan Criteria who underwent percutaneous cryoablation. To our best knowledge, this is the largest patient cohort in this type of study. Our study profiled a number of fundamental clinical features of percutaneous cryoablation in the treatment of HCC and may substantially update the current concept of cryoablation in HCC.

Several recent studies reported that after RFA treatment for small HCC, the CR rates, major complications and treatment-related deaths were 94.7–99.4%, 1.0–2.2%, and 0–0.3%, respectively[19–21]. In the present study, using similar patient population and study design, we demonstrated that percutaneous cryoablation resulted in 97.2% of CR rates, 2.8% major complications and no treatment-related deaths in treating patients with HCC within Milan criteria. Furthermore, as another critical validating parameter of PLATs, LR was observed in 11.9% of our patients after a median follow-up of 30.1 months, which also matched the reported range of LR rate following RFA (from 2.1 to 16.4%)[22]. Therefore, although our study is an observational one with no control, the results indicated the safety, efficacy and local tumor control following cryoablation appear comparable to those following RFA. Indeed, the above postulation was recently verified by our multiple-center randomized controlled trial (RCT), where the safety, efficacy and 5-year OS of cryoablation and RFA were compared. The results demonstrated that both modalities gave equally good outcomes in the treatment of HCCs ≤ 4 cm, except that cryoablation resulted in a significantly lower LR rate than that of RFA[14]. Interestingly, the lower LR rate of cryoablation was also reported by another Japanese team recently[13].

As a result of the epidemic of HBV in China, HBV-related cirrhosis and HCC presented in younger age has become an important clinical issue. In our study, the 5% youngest patients (42 of them, aged from 18 to 36, all had childhood HBV infection) carried significantly dismal prognosis with a median OS of 46.6 months, compared to 76.7 months of the relatively older patients. Therefore, contrary to the data elsewhere [19,23,24], younger age in Chinese patients with HBV-related HCC might be a predictor of poor prognosis. Our analysis further demonstrated the baseline HBV DNA load > 10^6^ copies/ml as an independent predictor for poor survival, together, these data again highlighted the critical role of timely and adequate anti-HBV therapies in the prevention and treatment of HCC[25].

Our analysis identified that repeated cryoablations to the same lesion was associated with local HCC recurrence. In our study, as did in the RFA studies[19–21], one or two repeat cryoablations were applied to HCCs that were failed to be eliminated by the initial ablation, however, multivariate analysis identified that even with primary CR, HCCs received a second or third cryoablation were 1.7 and 4.7 times more likely to develop LR than those received only once. Therefore, HCC seemed to be resistant to repeat cryoablations if it failed to be completely ablated after the first treatment attempt, nevertheless, it remains to be determined whether currently widely accepted one or two iterative ablation protocol brings more harm or benefit before further RCTs.

Cryoshock is a rare, but fatal cryoablation-related complication. According to a survey study, cryoshock occurred in about 1% patients who underwent hepatic cryosurgery[17], but the exact rate of cryoshock in percutaneous cryoablation remains unknown. Although cryoshock was once considered as a major obstacle to further application of cryoablation for HCC, in the present study, cryoshock did not occur in our 1401 cryoablation sessions. Moreover, even in our life-time experience in treating > 2000 patients with cryoablation, cryoshock occurred in only one case[26]. Therefore, in the era of modern percutaneous cryoablation, cryoshock can be an extremely rare complication. In the present study, cryoreaction was observed in about 0.8% of the patients, all ended with good outcome without mortality. The mechanisms by which patients’ reaction to cryoablation-induced necrotic cells are complicated, probably the essence of either cryoshock or cryoreaction is a tumor lysis-related inflammatory response (TLIR) in various extent. It is now widely accepted that the presence and severity of TLIR are largely dependent upon the volume of the destroyed tumor tissue[27,28]. Thus, the crux of avoiding cryoshock might be to set a limit to the targeted tumor volume in each cryoablation therapy. As demonstrated in our study, complete ablation of a single HCC ≤ 5 cm with percutaneous cryoablation on one occasion should be generally safe.

We use both CT and US for guidance in our cryoablation. CT is used to guide cryo-probe into the desired tumor, after that, US is used to monitor the size and the margin of the ice ball formed in the tumor. Although technically, MRI should be the best guidance method, but it is too expensive and requires special non-metal surgical instruments. CT is the second best, and the most feasible one. Compare to MRI, CT is affordable and is compatible for the routine surgical instruments. Compared to US, CT provides images of significant higher resolution which can give very clear organ structures during the inserting of probes.

The effect of cryoablation was generally checked 1 week after the treatment by enhanced CT or MRI scan, this is for the followings: 1) the ablated tumor needs time to underwent necrosis or apoptosis, before that, a radiographic tumor-like lesion could be residual tumor or inflammatory reaction, 1 week is an appropriate time point according to our experiences; 2) kidney is the mostly affected organ by the tumor lysis after cryoablation, thus contrast media used in enhanced CT or MRI should be avoided right after cryoablation because it may raise the risk of acute renal injury.

Although cryoablation has not been included to the current treatment algorithms for HCC, growing studies have well demonstrated the favoring outcomes of this ablation therapy [11–14]. According to our experiences, in most of the current clinical circumstances, cryoablation can give comparable outcomes to that of RFA, and the two therapies may thus be interchangeable in treating HCC within Milan criteria. We will preferably suggest considering cryoablation in the following contexts: 1) when tumors are closed to vital organs or vessels where elegant control of the ablative margin is required. This is because cryoablation can create more precise ablative zone when guided by US, and even more so by CT or MRI that is not generally available with other PLATs including RFA; 2) since cold is a natural attenuater of pain, cryoablation rarely needs general anesthesia and consumes less analgesics. This property is helpful when patient is in weak constitution or with tumors located near hepatic capsule or diaphragm, which usually cause overt pain after RFA[7]; 3) cryoablation is the first, or maybe the only, option of ablative therapy for patients with implanted electronic medical devices or metal materials because it does not disturb or create electronic loops. However, these indications still need to be further validated. In addition, cryoablation may also hold advantages in the medical costs over RFA. In our hospital, the average cost of cryoablation including the maintenance of equipments is approximately 40% cheaper than that of RFA.

There were several limitations of our study. First, this was a retrospective, single-arm study carried out in a single center. Clearly, more multiplecenter RCTs comparing cryoablation with other ablation modalities, such as RFA, were needed to further confirm our findings. Second, we used the current standard definition to determine LR[18]. However, it should be noted that this definition may be limited in differentiating true LR from new HCC occurrences abutting the margin of the cryoablation zone. This might have contributed to higher rates of LR in our study. Third, the underlying cirrhosis and HCC in our patients were majorly diagnosed based on clinical or imaging presentations, but not histology.

In conclusion, by using a large and long-term followed cohort, we demonstrated that cryoablation is an effective and safe ablation modality for the treatment of cirrhotic patients with HCC within Milan criteria. The short- and long-term outcomes and complication rates of cryoablation were comparable to that of the reported RFA studies. Considering the additional unique advantages it endows, cryoablation could be an important complement to the current thermal-dominated ablative techniques and should be considered as one of the standard ablation modalities for HCC.
